# Supplementary material for: The genome sequence of the Greater sandeel, Hyperoplus immaculatus (Corbin, 1950) (Uranoscopiformes: Ammodytidae)
Source: Wellcome Open Res. 2025 Nov 5;10:622. [Version 1] doi: 10.12688/wellcomeopenres.25072.1 (PMC12745857; doi:10.12688/wellcomeopenres.25072.1)
Supplement: Peer review history

# Reviewer report: Reviewer response for version 1

**DOI:** 10.21956/wellcomeopenres.27639.r140516

**Published:** 2025-12-29

**Authors:** David Barros-García

**Affiliations:** 1 Centro Interdisciplinar de Investigação Marinha E Ambiental (CIIMAR/CIMAR), Matosinhos, Portugal

The proposed article presents the first genome of the species Hyperoplus immaculatus (Corbin, 1950). To this end, the authors have used a set of techniques that includes PacBio reads and HiC libraries. The genome obtained is of normal size for a Hyperoplus immaculatus (Corbin, 1950) fish and has shown good quality indicators (97.6% BUSCO) and 92.28% of the genome assigned to 24 chromosome-level scaffolds.

 I have no objections to the technical aspects of the manuscript, but I do feel that some lines comparing the results with similar genomes are missing, as well as the usefulness of this genome for other studies (conservation, evolution, biogeography, etc.).

Are sufficient details of methods and materials provided to allow replication by others?

Yes

Is the rationale for creating the dataset(s) clearly described?

Yes

Are the datasets clearly presented in a useable and accessible format?

Yes

Are the protocols appropriate and is the work technically sound?

Yes

Reviewer Expertise:

My research focuses on integrated taxonomy, population genetics and evolutionary biology.

I confirm that I have read this submission and believe that I have an appropriate level of expertise to confirm that it is of an acceptable scientific standard, however I have significant reservations, as outlined above.

# Reviewer report: Reviewer response for version 1

**DOI:** 10.21956/wellcomeopenres.27639.r140515

**Published:** 2025-12-11

**Authors:** Xizhao Zhang

**Affiliations:** 1 Freshwater Fisheries and Germplasm Resources Utilization, Wuxi, China

This research presents a high-quality chromosome-level genome assembly for *Hyperoplus immaculatus*. The assembly utilizes PacBio HiFi sequencing reads, constructs scaffolds using Hi-C data, and undergoes annotation via RNA sequencing data. The primary assembly data covers 797.79 Mb of sequence, comprising 1,567 scaffolds. Of this, 91.28% of the sequence was assembled into 24 chromosome pseudomolecules, along with a 16.53 kb mitochondrial genome. This represents the second genome assembly for the genus *Hyperoplus* and will deepen our understanding of the sand eel's ecology—a species of significant research value as a key prey fish for marine predators.

 Regarding two suggestions for this paper:

 1: In the Genome assembly and Assembly quality assessment sections, the authors detail the software and pipeline used but lack descriptions of specific software parameters. If default parameters were used, please state this.

 2: The genome report section lacks a description of the mitochondrial genome; it is recommended to add this.

Are sufficient details of methods and materials provided to allow replication by others?

Yes

Is the rationale for creating the dataset(s) clearly described?

Yes

Are the datasets clearly presented in a useable and accessible format?

Yes

Are the protocols appropriate and is the work technically sound?

Yes

Reviewer Expertise:

Fisheries Resources，Genomics

I confirm that I have read this submission and believe that I have an appropriate level of expertise to confirm that it is of an acceptable scientific standard.

# Reviewer report: Reviewer response for version 1

**DOI:** 10.21956/wellcomeopenres.27639.r139621

**Published:** 2025-11-25

**Authors:** Nick Jeffery

**Affiliations:** 1 Bedford Institute of Oceanography, Fisheries and Oceans Canada, Canada, Canada

This article provides a summary of the greater sandeel genome, assembled using PacBio reads, HiC libraries, and RNA sequencing. The genome was assessed to be approximately 97% complete based on the estimated genome size and BUSCO scores. The paper is technically sound, but I do have the following questions:

 Did the authors assemble a transcriptome from the RNA reads, or was the RNAseq just used for annotation through the Ensembl pipeline? 

 Could the authors provide a summary of coding vs non-coding proportions of the genome, including transposable element content? Software such as Earl Grey has made transposable element identification quite simple, and is useful information for genome evolution studies? 

 Does the number of pseduo-chromosomes match what is expected for this family/genus? It appears so, so the authors could cite a reference on this (e.g., Ocalewicz et al. 2019 Mar Biol Research)

 A short paragraph or sentence at the end of the manuscript could help state the importance of this species. Are additional population genetics studies being conducted on this species or is there a specific conservation focus? I recognize the short nature of these data reports, but a short rationale on why this species was chosen for genome assembly would be useful.

Are sufficient details of methods and materials provided to allow replication by others?

Yes

Is the rationale for creating the dataset(s) clearly described?

Yes

Are the datasets clearly presented in a useable and accessible format?

Yes

Are the protocols appropriate and is the work technically sound?

Yes

Reviewer Expertise:

My research focuses on aquatic genomics, conservation biology, and evolutionary biology. I specifically focus on population genomics of marine organisms, and genome assembly and evolution. My research also uses environmental DNA metabarcoding for biodiversity monitoring in the marine realm.

I confirm that I have read this submission and believe that I have an appropriate level of expertise to confirm that it is of an acceptable scientific standard, however I have significant reservations, as outlined above.
